# The Bird That Sung in May

**Published:** 1864-01

**Authors:** 


### THE BIRD THAT SUNG IN MAY.

A BIRD last spring came to my window-shutter,
One lovely morning at the break of day;
And from his little throat did sweetly utter
A most melodious lay.

He had no language for his joyous passion,
No solemn measure, no artistic rhyme;
Yet no devoted minstrel e'er did fashion
Such perfect tune and time.

It seemed of thousand joys a thousand stories,
All gushing forth in one tumultuous tide;
A hallelujah for the morning-glories
That bloomed on every side.

And with each canticle's voluptuous ending,
He sipped a dew-drop from the dripping pane;
Then heavenward his little bill extending,
Broke forth in song again.

I thought to emulate his wild emotion,
And learn thanksgiving from his tuneful tongue;
But human heart ne'er uttered such devotion,
Nor human lips such song.

At length he flew, and left me in my sorrow,
Lest I should hear those tender notes no more;
And though I early waked for him each morrow,
He came not nigh my door.

But once again, one silent summer even,
I met him hopping in the new-mown hay;
But he was mute, and looked not up to heaven—
The bird that sung in May.

Though now I hear from dawn to twilight hour
The hoarse woodpecker and the noisy jay,
In vain I seek through leafless grove and bower
The bird that sung in May.

And such, methinks, are childhood's dawning pleasures,
They charm a moment and then fly away;
Through life we sigh and seek those missing treasures,
The birds that sung in May.

This little lesson, then, my friend, remember,
To seize each bright-winged blessing in its day;
And never hope to catch in cold December,
The bird that sung in May.

